# The G-Protein-Coupled Long-Chain Fatty Acid Receptor GPR40 and Glucose Metabolism

**DOI:** 10.3389/fendo.2014.00152

**Published:** 2014-09-26

**Authors:** Tsutomu Tomita, Kiminori Hosoda, Junji Fujikura, Nobuya Inagaki, Kazuwa Nakao

**Affiliations:** ^1^Department of Diabetes, Endocrinology and Nutrition, Kyoto University Graduate School of Medicine, Kyoto, Japan; ^2^Medical Innovation Center, Kyoto University Graduate School of Medicine, Kyoto, Japan

**Keywords:** GPR40, FFAR1, LCFA, insulin secretion, pancreatic beta cells

## Abstract

Free fatty acids (FFAs) play a pivotal role in metabolic control and cell signaling processes in various tissues. In particular, FFAs are known to augment glucose-stimulated insulin secretion by pancreatic beta cells, where fatty acid-derived metabolites, such as long-chain fatty acyl-CoAs, are believed to act as crucial effectors. Recently, G-protein-coupled receptor 40 (GPR40), a receptor for long-chain fatty acids, was reported to be highly expressed in pancreatic beta cells and involved in the regulation of insulin secretion. Hence, GPR40 is considered to be a potential therapeutic target for the treatment of diabetes. In this review, we summarize the identification and gene expression patterns of GPR40 and its role in glucose metabolism. We also discuss the potential application of GPR40 as a therapeutic target.

## Introduction

Free fatty acids (FFAs) are essential nutrients that also act as signaling molecules in various tissues. Long-chain fatty acids (LCFAs) play a role in the augmentation of glucose-stimulated insulin secretion (GSIS) (1). GSIS was observed to be considerably decreased by FFA depletion following *in vivo* administration of nicotinic acid to rats (2) and humans (3). Thus, FFA-mediated augmentation is considered to be physiologically significant. However, the underlying mechanisms of FFA-mediated augmentation of GSIS have not been fully elucidated. Several investigators have recently demonstrated that FFAs act as ligands for membrane-bound G-protein-coupled receptors (GPCRs) such as G-protein-coupled receptor 40 (GPR40), GPR41, GPR43, and GPR120. Among these, GPR40 is preferentially expressed by pancreatic beta cells in rodents and augments GSIS after acute exposure to LCFAs, highlighting the role of GPR40 as a potential key molecule in the regulation of insulin secretion.

## LCFA Receptor GPR40

GPR40 consists of 300 residues and was originally reported as an orphan GPCR (4). GPR40 was deorphaned by screening using a fluorometric imaging plate reader (FLIPR) system, which detects increases in Ca^2+^ concentrations in cultured cells with transiently expressed GPR40 cDNA (5, 6). GPR40 is reportedly activated by LCFAs (C12–22) and several eicosanoids in theoretically physiological concentration ranges. The profiles of putative GPR40 ligands are well conserved among mice, rats, and humans (5).

## GPR40 Gene Expression in Rodents

Among rat tissues, GPR40 mRNA is almost exclusively expressed in the pancreas. In pancreatic islets, GPR40 mRNA levels were found to be approximately 17-fold higher than the levels in the pancreas, suggesting selective GPR40 expression by pancreatic islets. Considerable amounts of GPR40 mRNA were detected in the pancreatic beta cell lines MIN6, betaTC-3, HIT-T15, and Rin5F but not in the pancreatic alpha cell line alphaTC1. Furthermore, *in situ* hybridization with rat pancreatic islets suggested that GPR40 mRNA is preferentially expressed in pancreatic beta cells (5).

Reports using anti-GPR40 antibodies suggest that GPR40 protein is also probably preferentially expressed in pancreatic islets (7, 8).

## Roles of GPR40 in Regulation of Insulin Secretion

In MIN6 cells, insulin secretion was augmented by LCFAs in a dose-dependent manner, and the augmentation was observed only under hyperglycemic conditions (11–22 mM) (5), indicating the LCFA-mediated augmentation of insulin secretion is glucose-dependent. Silencing of GPR40 gene expression using siRNA almost abolished the augmentation effects of LCFAs, indicating that GPR40 is involved in LCFA-mediated regulation of insulin secretion. GPR40 is a class A GPCR, highlighting the potential of GPR40 as a target for novel anti-diabetic oral drugs with low risk of hypoglycemia, considering that LCFA-mediated augmentation of insulin secretion is glucose-dependent.

## GPR40 Gene Expression in Humans

Although GPR40 is reportedly preferentially expressed by pancreatic beta cells in both rats and mice, little is known about GPR40 gene expression in humans. In this context, we assessed GPR40 mRNA expression in fresh human tissues obtained during surgery (9, 10). Analysis of 12 specimens of non-tumor pancreatic tissues revealed a considerable amount of GPR40 mRNA in each. In three pancreatic islet tissues specimens, GPR40 mRNA levels were approximately 20-fold higher than those in pancreatic tissues, comparable to the levels of sulfonylurea receptor 1, which is known to be highly expressed in pancreatic beta cells. High levels of GPR40 mRNA were detected in insulinoma (beta cell tumor) tissues in three cases; in contrast, GPR40 mRNA was undetectable in glucagonoma (alpha cell tumor) tissues (10, 11). In human pancreas, GPR40 mRNA level is positively and significantly correlated with the insulinogenic index, an index reflecting the function of pancreatic beta cells. These results indicate that GPR40 is highly expressed in human pancreatic beta cells and possibly involved in the positive regulation of insulin secretion (10).

## Regulation of GPR40 Gene Expression

Though the mechanisms underlying the regulation of GPR40 gene expression is not fully understood, possible mechanisms include the PDX-1/IPF1 (12), which reportedly binds to the promoter region of the GPR40 gene (13). Moreover, nutrients and therapeutic drugs such as glucose (12), palmitate, and rosiglitazone (8) are reportedly involved in the regulation of GPR40 gene expressions.

## Therapeutic Implications of GPR40

Although an initial report of systemic GPR40 knockout (KO) mice and beta cell-specific GPR40 transgenic (Tg) mice using the PDX-1/IPF1 promoter suggested possible involvement of GPR40 in insulin resistance in the liver and beta cell failure (14), later reports using GPR40 KO mice found no link between GPR40 and beta cell dysfunction (15, 16). Studies using GPR40 KO mice suggest the implication of GPR40 in the regulation of insulin secretion, at least under some conditions including loading of intralipid (17), high-fat diet (15), hyperglycemic glucose clamp, and arginine (18). Furthermore, GPR40 Tg mice with the mouse INS2 promoter exhibited better glucose tolerance with enhanced GSIS (19), suggesting therapeutic implications of GPR40 rather than a gateway of beta cell toxicity.

Additionally, recent reports suggest that GPR40 is expressed in enteroendocrine cells and involved in the positive regulation of intestinal hormones including glucagon-like peptide-1 (GLP-1), glucose-dependent insulinotropic polypeptide (GIP), and cholecystokinin (20–22).

## GPR40 Agonists as Anti-Diabetic Drugs

Recently, TAK-875 (Fasiglifam), a novel GPR40 selective agonist (23), was reported as a potential oral anti-diabetic drug. The potency of TAK-875 is approximately 400-fold greater than that of the endogenous ligand oleic acid (24), and it does not activate GPR120 (23), another GPCR for LCFAs. TAK-875 augmented insulin secretion under high-glucose conditions in the rat pancreatic beta cell line INS1 833/14 (24) and human pancreatic islets (25) but did not affect glucagon secretion in humans (25), in accordance with the observations in humans by our group and others (9–11). TAK-875 significantly improved glycemic control with the augmentation of insulin secretion in diabetic rat models such as Wistar fatty rats (23) and Zucker diabetic fatty rats (24).

In phase 2, randomized, double-blind, placebo-controlled trial in patients with type 2 diabetes, HbA1c was decreased in a dose-dependent manner in TAK-875 groups, and the HbA1c-lowering effect (50–200 mg, approximately −1.1% in 12 weeks) was comparable to that in glimepiride (4 mg) group, while the incidence of hypoglycemia in TAK-875 was similar to the placebo group and markedly lower than the glimepiride group (26). In Japanese patients with type 2 diabetes, 12-week treatment with TAK-875 also decreased HbA1c levels in a dose-dependent manner, and the HbA1c-lowering effect (50–200 mg, approximately −1.3%) was comparable to that in the glimepiride (1 mg) group (27).

Though TAK-875 seemed to be a promising anti-diabetic drug, regrettably, its development was terminated in 2013 because of the risk of possible liver damage. Although the cause of the liver damage remains unclear, GPR40 is not expressed in the human liver (6, 10), suggesting that the toxicity may not be due to the GPR40 receptor itself but chemical characteristic of TAK-875 or its dose used in the clinical trials. Still, several GPR40 agonists continue to be evaluated in both preclinical (Bristol-Myers Squibb, Merck, Amgen, Johnson & Johnson, Astellas, Daiichi Sankyo, Piramal, and Connexios) and clinical (Japan Tobacco) trials, and the further development is expected in the study elucidating the significance of GPR40 in glucose and other metabolism.

## Conclusion

**Figure 1:**
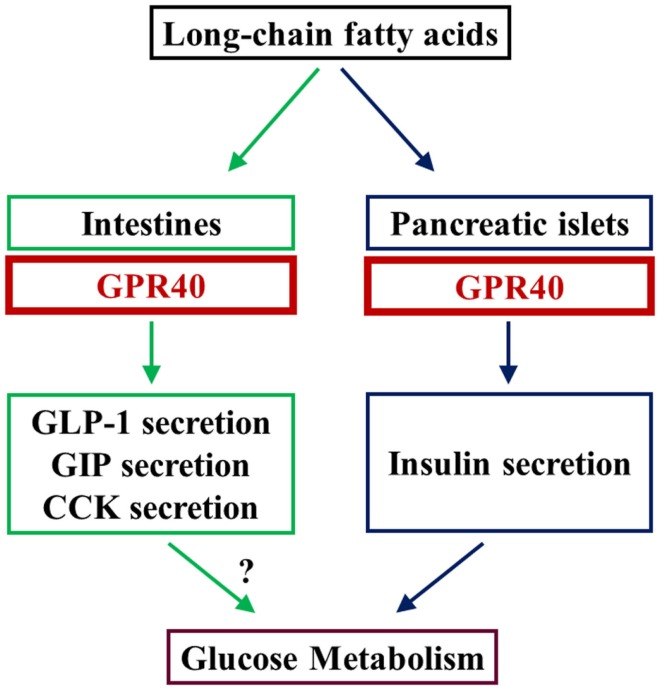
**LCFAs augment insulin secretion by stimulating GPR40 in beta cells in the pancreas**. GPR40 is also reportedly involved in the positive regulation of secretion of some intestinal hormones. Studies using GPR40 agonists and analyses of beta cell-specific GPR40 transgenic mice indicate that GPR40 is involved in the regulation of glucose metabolism, at least by the augmentation of insulin secretion. Because LCFA-mediated augmentation of insulin secretion is glucose-dependent, GPR40 is thought to be a potential target for novel anti-diabetic drugs with low risk of hypoglycemia.

Incretin mimetic-type drugs have been implicated in GPCR-mediated regulation of insulin secretion in diabetes. GPR40 is a GPCR that is highly expressed in pancreatic beta cells and involved in insulin secretion in rodents and humans. Hence, GPR40 is a potential therapeutic target in diabetes, which can lead to the development of oral drugs with fewer hypoglycemic side effects. Furthermore, GPR40 is reportedly implicated in the regulation of incretin secretion from enteroendocrine cells. GPR40 may be important to unveil the link between FFA signaling and beta cell function as well as glucose metabolism (Figure 1). Hence, further studies are warranted to elucidate the physiological and pathophysiological implications of GPR40.

## Conflict of Interest Statement

The authors declare that the research was conducted in the absence of any commercial or financial relationships that could be construed as a potential conflict of interest.
